# Supplementary material for: Neuronal parts list and wiring diagram for a visual system
Source: Nature. 2024 Oct 2;634(8032):166–80. doi: 10.1038/s41586-024-07981-1 (PMC11446827; doi:10.1038/s41586-024-07981-1)
Supplement: Supplementary file 5 — Cards for all cell types. Each card has 6 panels (clockwise): logical connectivity predicate diagram, numeric stats, illustration of a typical cell, followed by 3D rendering of all cells from three orthogonal viewpoints. Also available at Codex. [file 41586_2024_7981_MOESM5_ESM.zip › pdf/Lat.pdf]

Lat

predicate precision: 60%, recall: 50%

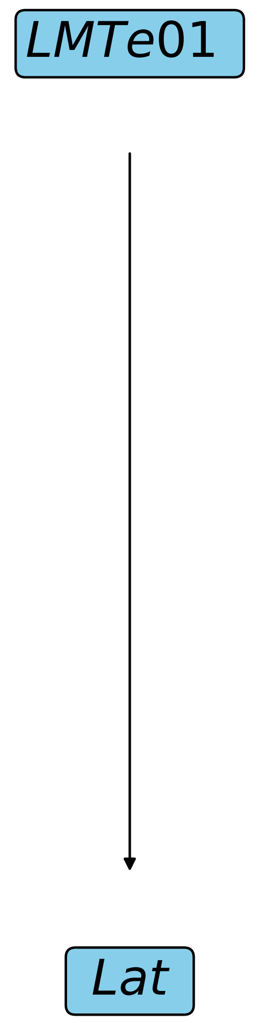

avg. stats

cells	6
NT prediction	-
top in/out regions	LO (46%) / ME (36%)
in/out degree	22 / 4
in/out synapses	96 / 12
cable len	$1,720\mu$
surface	$3,315\mu^2$
volume	$232\mu^3$

morphology

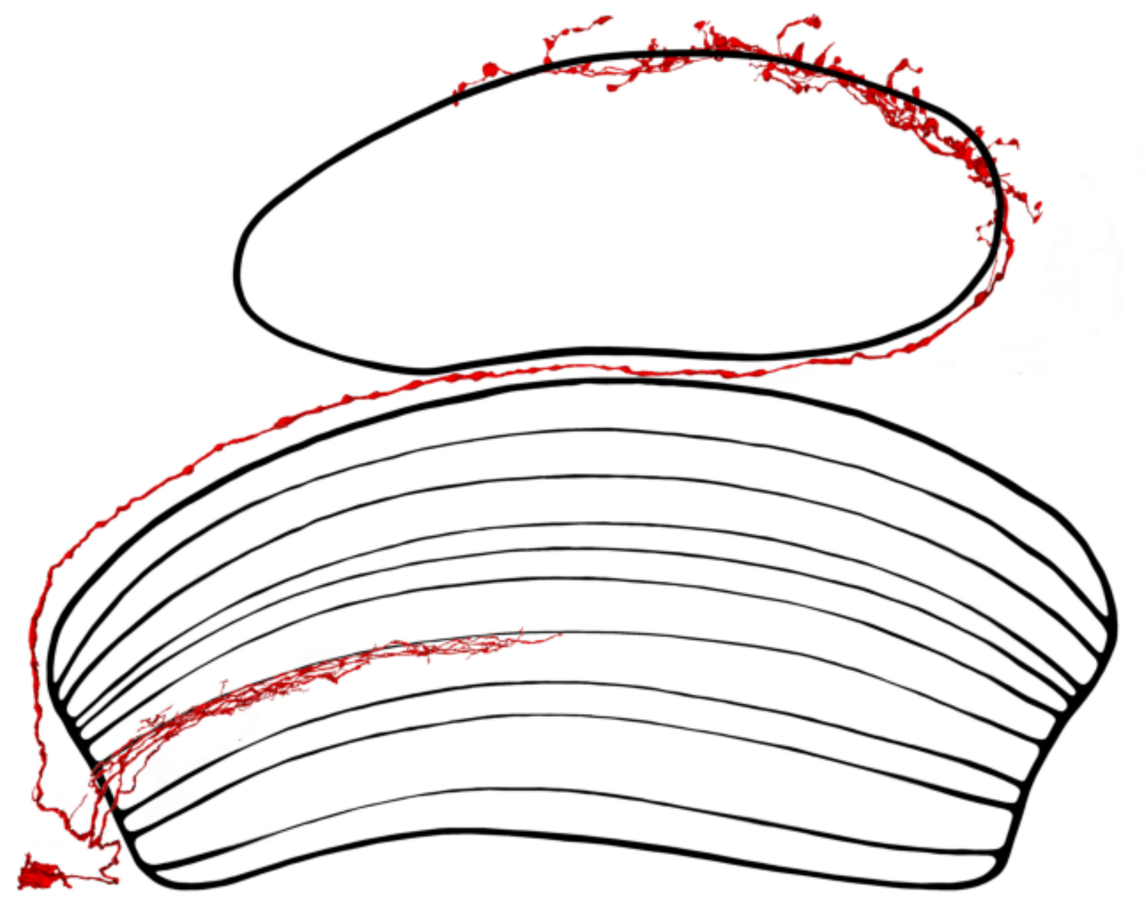

anterior view

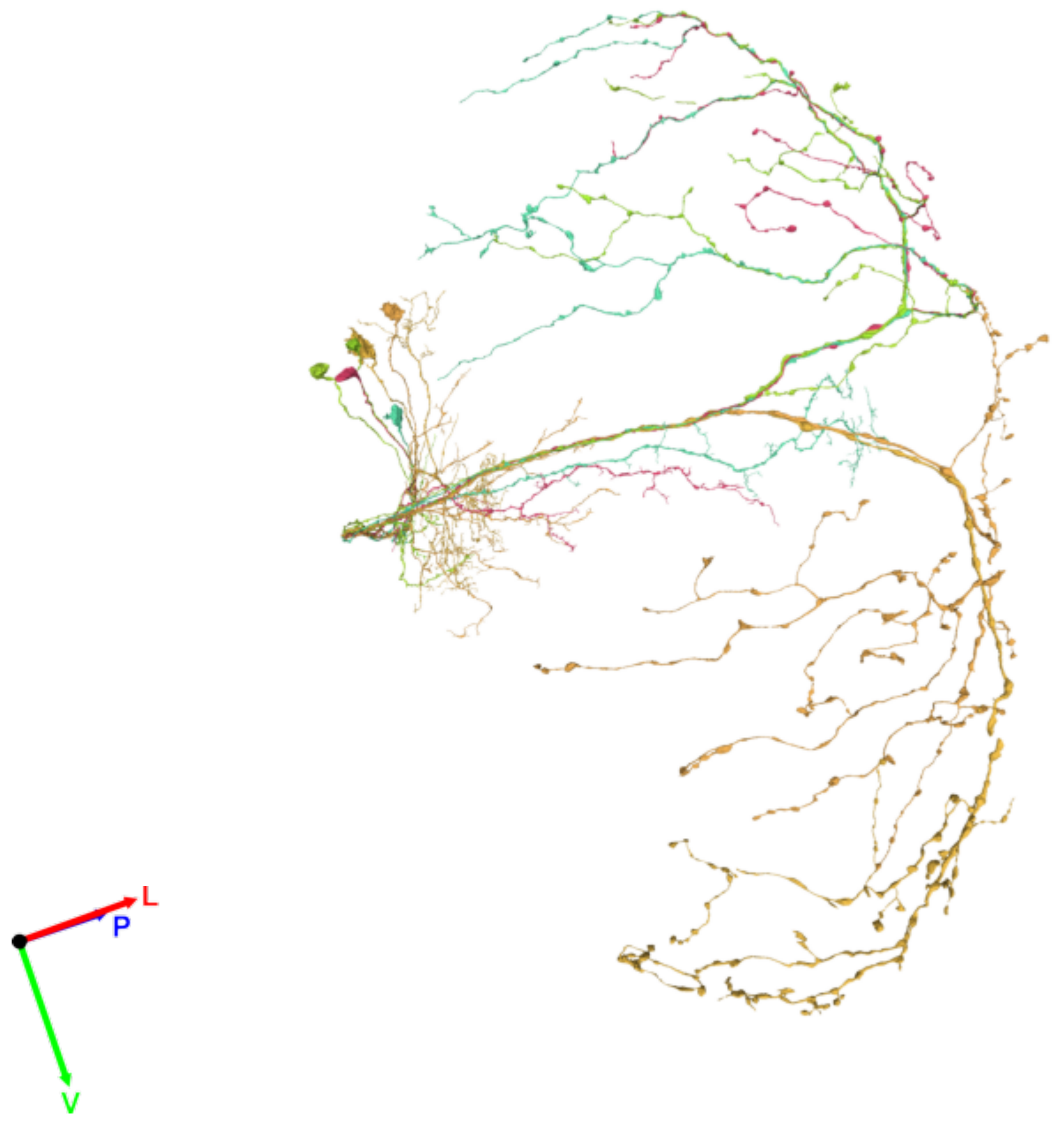

ventral view

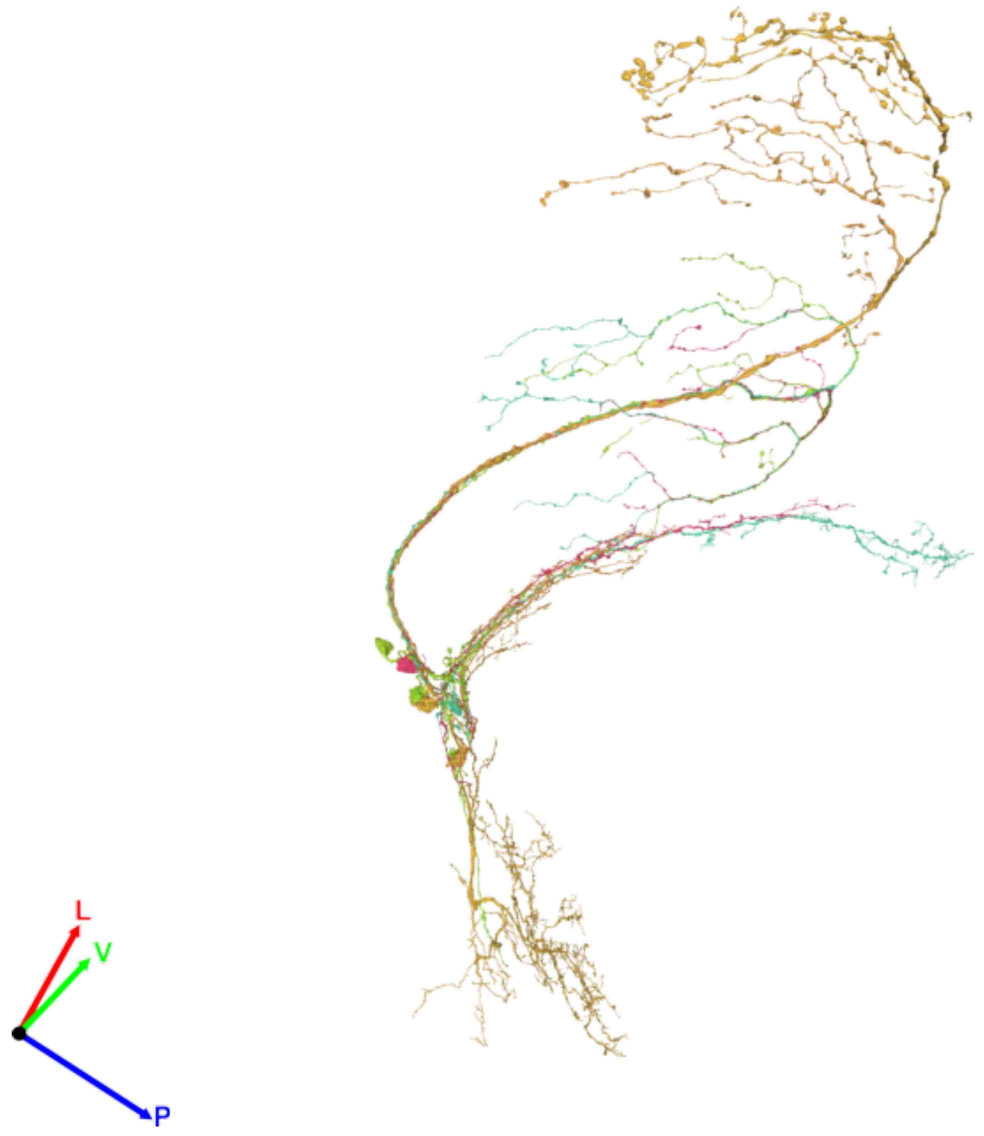

side view

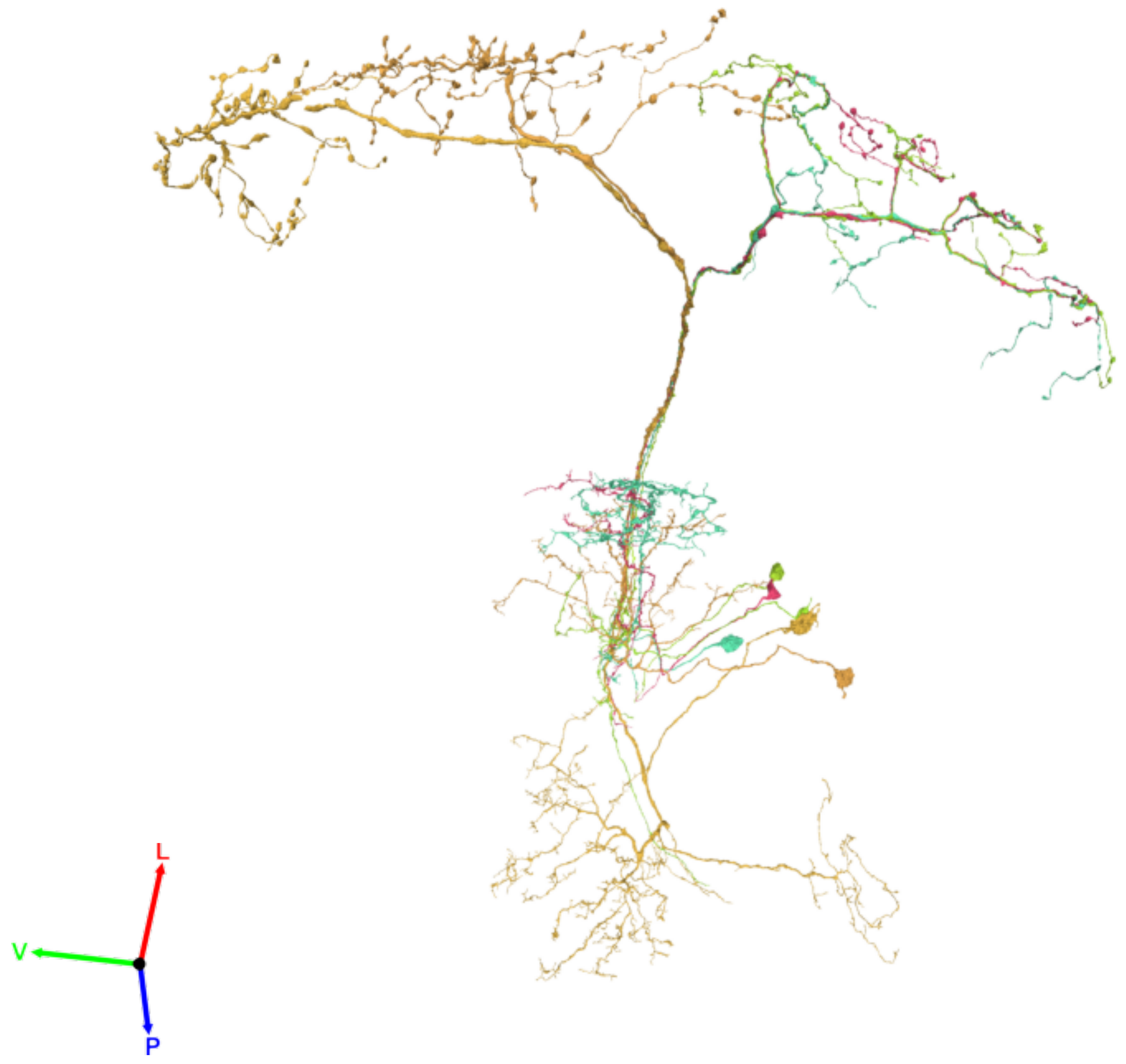

Lamina Tangential
